# Reduced expression of a gene proliferation signature is associated with enhanced malignancy in colon cancer

**DOI:** 10.1038/sj.bjc.6604560

**Published:** 2008-08-26

**Authors:** A Anjomshoaa, Y-H Lin, M A Black, J L McCall, B Humar, S Song, R Fukuzawa, H-S Yoon, B Holzmann, J Friederichs, A van Rij, M Thompson-Fawcett, A E Reeve

**Affiliations:** 1 Department of Biochemistry and Cancer Genetics Laboratory, University of Otago, Dunedin, New Zealand; 2 Department of Surgery, University of Auckland, Auckland, New Zealand; 3 Department of Pathology, University of Otago, Dunedin, New Zealand; 4 Department of Surgery, Technische Universität München, München, Germany; 5 Department of Medical and Surgical Sciences, University of Otago, Dunedin, New Zealand

**Keywords:** proliferation signature, colon cancer, disease-free survival

## Abstract

The association between cell proliferation and the malignant potential of colon cancer is not well understood. Here, we evaluated this association using a colon-specific gene proliferation signature (GPS). The GPS was derived by combining gene expression data obtained from the analysis of a cancer cell line model and a published colon crypt profile. The GPS was overexpressed in both actively cycling cells *in vitro* and the proliferate compartment of colon crypts. K-means clustering was used to independantly stratify two cohorts of colon tumours into two groups with high and low GPS expression. Notably, we observed a significant association between reduced GPS expression and an increased likelihood of recurrence (*P*<0.05), leading to shorter disease-free survival in both cohorts. This finding was not a result of methodological bias as we verified the well-established association between breast cancer malignancy and increased proliferation, by applying our GPS to public breast cancer data. In this study, we show that reduced proliferation is a biological feature characterizing the majority of aggressive colon cancers. This contrasts with many other carcinomas such as breast cancer. Investigating the reasons underlying this unusual observation may provide important insight into the biology of colon cancer progression and putative novel therapy options.

Defective regulation of cell proliferation is a fundamental feature of cancer. By providing a genome-wide insight, microarray technology has revealed that conserved tumour expression patterns include many proliferation-associated genes termed as ‘proliferation signatures’. In most cancers, an increased expression of proliferation signatures has been associated with enhanced malignancy (Rosenwald *et al*, 2003; Krasnoselsky *et al*, 2004; Dai *et al*, 2005) suggesting cell proliferation as a driving force for cancer progression. Breast cancer is a typical example where the hyper-proliferative nature of more aggressive subtypes has been confirmed by array-based proliferation assays (Sorlie *et al*, 2001; Dai *et al*, 2005). These studies have also proved the superiority of cell proliferation analysis by microarrays over conventional methods regarding its objectivity and because of the assessment of multiple genes (Paik *et al*, 2004; Glinsky *et al*, 2005; Whitfield *et al*, 2006).

In colorectal cancer (CRC), the impact of tumour proliferation rate on malignancy is unclear because the conventional proliferation markers have produced conflicting results (Supplementary Table 1). To date, no proliferation signature has been established to address such an association.

To investigate the association between proliferation and aggressiveness of colon cancer, we designed a colon-specific gene proliferation signature (GPS) that was derived from common expression patterns of an *in vitro* CRC model, and of human colon crypt compartments as published by Kosinski *et al* (2007). The GPS was applied to evaluate two independant cohorts of colonic tumours and revealed an inverse relationship between tumour cell proliferation and unfavourable clinico-pathological parameters.

## Materials and methods

### Cell cultures

The following 10 CRC cell lines were used: DLD-1, HCT-8, HCT-116, HT-29, LoVo, Ls174T, SK-CO-1, SW48, SW480 and SW620 (ATCC, Manassas, VA, USA). Cells were cultivated in α-MEM supplemented with 10% foetal bovine serum, 100 IU ml^−1^ penicillin and 100 *μ*g ml^−1^ streptomycin (GIBCO-invitrogen, CA, USA). Two cell cultures were established for each cell line for RNA preparation. The first culture was harvested at semi-confluence (50–60%) when cells were actively cycling. Cells in the second culture were harvested 48 h after reaching full confluence, when cells were slow-growing. A replicate experiment was performed to obtain RNA for dye-reversed hybridisations.

### Patients

Tumours from two cohorts of colon cancer patients were analysed. Patient follow-up was a minimum of 5 years. Disease-free survival (DFS) was calculated from surgery to the date of distant disease recurrence. Patients with rectal cancer, patients for whom the date of recurrence was unknown or received pre-operation adjuvant therapy were not included in this study.

Cohort A consisted of 108 New Zealand patients, who underwent surgery at Dunedin and Auckland hospitals between 1995 and 2000, and included all disease stages. Thirty patients received 5-FU-based post-operative adjuvant chemotherapy. Ethical approval was obtained from the Ethics Committee, Otago University.

Cohort B consisted of a group of 37 stage II German patients, who underwent surgery at the Technical University of Munich between 1995 and 2001. None of the patients received adjuvant therapy. Clinico-pathologic variables of both cohorts are summarised in Table 1.

### Array preparation and gene expression analysis

#### Cohort A tumours and cell lines

Gene expression profiling of cohort A tumours and cell lines was performed using arrays spotted with the MWG 30 K Oligo Set (MWG Biotech, NC, USA). RNA was extracted from cell lines and fresh-frozen tissues, using Tri-Reagent (Progenz, Auckland, NZ) and purified using RNeasy mini columns (Qiagen, Victoria, Australia). Ten micrograms of total RNA were oligo-dT primed and cDNA synthesis was carried out in the presence of aa-dUTP and Superscript II RNase H-Reverse Transcriptase (Invitrogen, CA, USA). Cy dyes were incorporated into cDNA using the indirect amino-allyl cDNA labelling method. A pooled RNA sample derived from a mixture of 12 cell lines was used as a reference for all hybridisations.

The Cy5-dUTP-tagged cDNA from an individual colorectal cell line or tissue sample was combined with Cy3-dUTP-tagged cDNA from the reference sample. The mixture was then purified using a QiaQuick PCR purification kit and co-hybridised to a microarray. Duplicate hybridisations for cell lines were performed following dye reversal.

After scanning with a GenePix 4000B microarray scanner (Axon, CA, USA), the foreground intensities from each channel were log_2_-transformed and normalised using the SNOMAD software (Colantuoni *et al*, 2002). Normalised values were collated and filtered using BRB-Array Tools version 3.6.0-β_3 (by Dr Richard Simon and Amy Peng Lam, National Cancer Institute). Genes of low signal intensity, or for which more than 20% of measurements across tissue samples or cell lines were missing, were excluded from further analysis.

#### Cohort B tumours

Total RNA was prepared from each tumour using the RNeasy mini kit (Qiagen, Hilden, Germany). Ten micrograms of total RNA were used to synthesise double-stranded cDNA with SuperScript II reverse transcriptase (Gibco-Invitrogen, NY, USA) and an oligo-dT-T7 primer (Eurogentec, Koeln, Germany). The biotinylated cRNA was synthesised from the double-stranded cDNA using the Promega RiboMax T7-kit (Promega, Madison, WI, USA) and Biotin-NTP labelling mix (Loxo, Dossenheim, Germany). The biotinylated cRNA was then purified and fragmented. The fragmented cRNA was hybridised to Affymetrix HGU133A GeneChips (Affymetrix, Santa Clara, CA, USA) and stained with streptavidin–phycoerythrin. The arrays were scanned with an HP-argon laser confocal microscope and the digitised image data were processed using the Affymetrix® microarray suite 5.0 software. Background correction and normalisation were performed in the R computing environment (Ihaka and Gentleman, 1996) using the robust multi-array average algorithm.

### Quantitative real-time PCR

The expression of seven randomly selected genes from the GPS (*MAD2L1*, *POLE2*, *CDC2*, *MCM6*, *MCM3*, *TOPK* and *GMNN*) was validated by quantitative real-time PCR (qRT–PCR) on an ABI Prism 7900HT Sequence Detection System using cell line cDNA and Taqman Gene Expression assays (Applied Biosystems, CA, USA). Relative fold changes were calculated using the 2^−ΔΔCT^ method (Livak and Schmittgen, 2001) with Topoisomerase 3A as the internal control. Reference RNA was used as the calibrator to enable comparison between different experiments.

### Immunohistochemical analysis

Ki-67 immunohistochemistry was performed on 40 cohort A tumours for which paraffin blocks were available, and an additional set of 33 rectal/rectosigmoid tumours was included to increase statistical power. Antigens were retrieved on 4-*μ*m sections in boiling citrate buffer (pH 6). Primary antibody (MIB-1, DakoCytomation, Denmark; dilution 1 : 50) was detected using the EnVision system (Dako EnVision) and the DAB substrate kit (Vector Laboratories, CA, USA). Five high-power fields were counted by two observers in a blinded manner. The Ki-67 proliferation index (PI) was presented for each tumour as the percentage of positively stained nuclei.

### Statistical analysis

The K-means clustering method was applied to stratify clinical samples on the basis of the GPS expression level using the TIGR MeV 4.0 software (Saeed *et al*, 2003). Using Pearson uncentered correlation, tumours from each cohort were assigned to two clusters (i.e., K-means clustering with *K*=2, 1000 iterations of clustering) with the threshold of occurrence in the same cluster set to 80%. The consensus clusters each contained tumours with similar GPS expression, resulting in two patient groups differing in their GPS levels. Either Fisher's exact test or the Kruskal–Wallis test was then used to evaluate the associations between the dichotomous GPS variable and clinico-pathologic parameters. Statistical analyses were performed using SPSS 15.0.0 (SPSS Inc., Chicago, IL, USA). For Ki-67 analysis, tumours were stratified into two clusters with the mean Ki-67 value as a cutoff point.

The gene set comparison function from the BRB-Array tools software was used to analyse the GPS for differential expression among subgroups defined by the clinico-pathologic variables. The GPS was considered to have a higher-than-expected number of genes differentially expressed (DE) between the classes being compared if the Kolmogorov–Smirnov (KS) resampling *P*-value was less than 0.005 (default value). The distribution of KS statistics was obtained by 100 000 iterations of the random resampling process.

Disease-free survival was plotted using the method of Kaplan and Meier, and a log-rank test was used to test for differences in survival time between defined clusters of patients according to the GPS or Ki-67 PI. A multivariate Cox proportional hazards model was developed using forward stepwise regression with predictive variables that were significant in the univariate analysis. Cox multivariate regression was not relevant for measuring the performance of the GPS in cohort B, as this population of tumours included only stage II colon cancers.

## Results

### Derivation of a GPS

**Figure 1:**
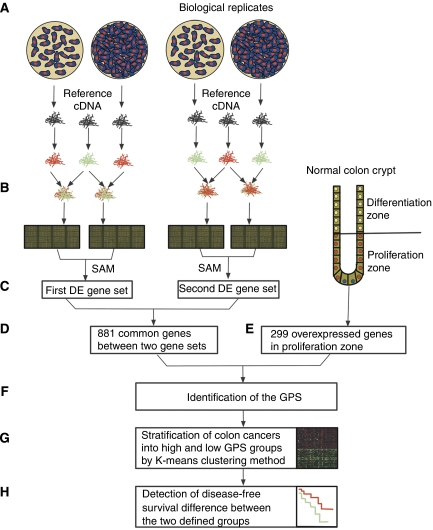
Overview methodology. A gene proliferation signature (GPS) was derived by combining the gene expression data from the analysis of a cell line model and colon crypts. (**A**) Ten colorectal cancer cell lines were cultured and harvested at semi-confluence and full confluence. Each cell culture was performed in duplicate. (**B**) Using a reference design, a mixture of sample and reference dye-labelled cDNAs was hybridised to a 30 K Oligo array. Dye orientation was reversed for biological replicates. (**C**) Statistical analysis of microarrays (SAM) was performed to identify differentially expressed (DE) genes between two stages of growth in cultures. Two gene sets were generated through the analysis of samples with identical dye labelling. (**D**) Only 881 genes that were presented in both SAM-generated gene sets and DE gene sets were selected. (**E**) Human colon crypt profiling resulted in identification of 299 DE genes with overexpression in the proliferation zone compared with the differentiation zone. (**F**) The GPS was generated by taking the overlapping genes between **D** and **E** gene sets. (**G**) Two cohorts of colon cancer patients were stratified into low and high groups according to the GPS expression using K-means clustering method. (**H**) Disease-free survival difference was calculated between the two defined groups.

To identify a set of genes whose expression was associated with tumour cell proliferative activity, two proliferation-based systems were analysed and integrated as described below and illustrated in Figure 1.

*A. Gene expression analysis of a CRC in vitro model*

The *in vitro* system involved identification of genes that were to reflect the proliferative activity of CRC cell lines. Genes DE between exponentially growing (nonconfluent) and growth-inhibited (confluent) cell lines were identified (Figure 1A–C). Firstly, DE genes between Cy5-labelled nonconfluent and confluent samples were identified by statistical analysis of microarray (two-class paired, FDR<1%; Tusher *et al*, 2001). Secondly, DE genes between Cy3-labelled nonconfluent and confluent samples (biological replicates) were identified using the same approach. To minimise gene-specific dye bias and other sources of variation, only genes that were present in both SAM-generated gene sets were selected. The gene set was further reduced to genes whose expression was consistently altered in the same direction in at least 8 out of 10 cell lines, yielding a total 881 DE genes with known annotation (Figure 1D).

Gene ontology (GO) analysis showed that categories related to cell cycle and DNA metabolism were the most over-represented biological themes among the DE genes, mainly because of genes that were overexpressed in exponentially growing cells (*P*<10^−5^).

*B. Gene expression profile of the proliferative compartment of colon crypts*

The second gene set used for the design of a GPS was based on the physiological expression profile of human colon crypts. Kosinski *et al* (2007) compared the proliferative bottom part of crypts with the differentiated crypt top, and identified 299 DE genes highly expressed in the proliferative bottom (Figure 1E). The GO terms that were over-represented within this gene list were related to cell proliferation and renewal, consistent with the physiological function of the bottom crypt compartment.

*C. Definition of the GPS*

To define a final GPS enriched in key-proliferative genes, the *in vitro* and *in vivo*-derived DE gene lists were screened for common genes (Figure 1F). Thirty-six genes were found to be overexpressed in both exponentially growing CRC cell lines and the proliferative crypt compartment. These genes were defined as GPS that included 15 cell cycle-related genes (Supplementary Table 2).

The expression of 7 genes randomly selected from the 15 cell cycle-related genes was validated by qRT–PCR on the cell line cDNAs. A close correlation between qRT–PCR and array data was observed (Supplementary Figure 1).

### Classification of colon tumours according to the GPS expression level

To investigate the association between the GPS and patient parameters, expression values of the GPS genes were first obtained from the array-generated expression profiles. Expression data were available for all 36 genes in cohort A, and 35 genes (except CDCA5) in cohort B tumours. For each cohort, tumours were split into two consensus clusters on the basis of their GPS expression using K-means clustering. Tumours with relatively high or low GPS expression were defined as having high or low proliferative activity, respectively (Figure 1G).

### Reduced expression of the GPS is associated with unfavourable clinico-pathologic variables

Intriguingly, we observed an association between *reduced* GPS expression and an increased risk of recurrence in both cohorts (Table 1). Groups with reduced GPS expression were significantly enriched for recurrent tumours (*P*=0.021 and 0.005 for cohort A and B, respectively). In cohort A, reduced GPS expression was also associated with higher disease stage (*P*=0.015). Further, reduced GPS expression was significantly more frequent in cohort A tumours with lymphatic invasion compared with those without lymphatic invasion (*P*=0.018). Gene set comparison analysis confirmed that GPS contained a higher-than-random proportion of DE genes among subgroups with clinico-pathologic parameters for which a significant association with the GPS was found in nonparametric tests (Supplementary Table 3).

### *Reduced* GPS expression is associated with *shorter* DFS in colon cancer

**Figure 2:**
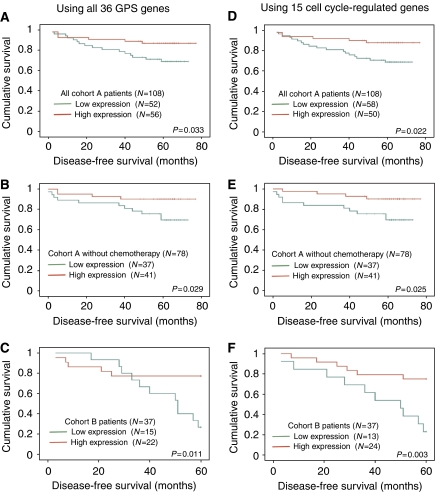
Disease-free survival analysis of colon cancer patients stratified into high and low groups according to the GPS expression or 15 cell cycle-regulated genes included in the GPS. In both cohorts, the low GPS groups had significantly shorter DFS compared with the high GPS groups (**A** and **C**). This difference was more significant when only cell cycle-regulated genes were used to stratify patients (**D** and **F**). The same association was found when the analysis was limited to those cohort A patients who received no adjuvant therapy (**B** and **E**).

To examine whether a difference in cell proliferation determined by the GPS may be associated with time to recurrence, DFS was plotted for low GPS and high GPS tumours (Figure 2). DFS was significantly shorter in patients with reduced GPS expression (*P*=0.033 and 0.011 for cohort A and B patients, respectively; Figure 2A and C). This association remained significant in cohort A, when patients with adjuvant therapy were excluded (*P*=0.029; Figure 2B).

If prolifetative activity is a crucial factor influencing DFS, then a GPS consisting of only genes directly implicated in cell cycle process should be sufficient to separate patients into groups with different survival rates. Therefore, the GPS was reduced to the 15 cell cycle-regulated genes and survival analysis was repeated. The modified GPS stratified patients into groups that differed in their DFS more stringently (*P*=0.022 and 0.003 for cohort A and B patients, respectively; Figure 2D–F) than the original GPS including all 36 genes. Therefore, colon cancer patients with tumours of lower proliferative activity are more likely to recur.

When the parameters predicting patient outcome in univariate analysis were investigated in a multivariate model, disease stage was the only independant predictor of 5-year DFS in cohort A (Table 2).

### *Increased* GPS expression is associated with *shorter* DFS in breast cancer

As the association between reduced GPS expression and poor colon cancer prognosis was an unexpected finding, we tested the validity of our GPS on public array data from two independant breast cancer cohorts. Using these data, an association between increased proliferation and bad outcome has been established earlier (van de Vijver *et al*, 2002; Pawitan *et al*, 2005).

**Figure 3:**
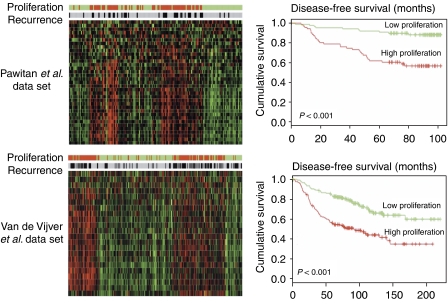
Stratification effect of the GPS on two cohorts of breast cancer patients. The heat maps represent the normalised gene expression values of the GPS genes across samples. Each row represents one gene and each column represents one sample. Colour bars on the top of heat maps represent proliferation groups (high proliferation is indicated in red, and low proliferation is indicated in green as defined by K-means clustering) and recurrence status (black, recurrence; grey, nonrecurrence). There is a close correlation between high GPS expression and recurrence in both cohorts. Disease-free survival is significantly shorter in the high GPS groups compared with the low GPS groups.

For each breast cancer data set, patients were stratified into two groups with either low or high GPS expression and their DFS was plotted. In both data sets, patients with increased GPS expression had significantly shorter DFS compared with patients with reduced GPS expression (*P*<0.0001; Figure 3), confirming the previously established association. Therefore, the association between low proliferation and bad colon cancer outcome is not the result of biased methodology.

### Ki-67 PI is not associated with outcome

Ki-67 PI ranged from 25 to 96%, with a mean value of 76.3±17.5 and a median value of 81.8%. When these 73 patients were stratified into two groups differing in their GPS expression, a significant difference in DFS was apparent (*P*=0.01; Supplementary Figure 2C). However, when patients were stratified into two groups according to the mean Ki-67 PI, the DFS of the group with a low PI was similar to that with a high PI (*P*=0.55; Supplementary Figure 2D). Furthermore, when analysis was limited to patients with the highest and the lowest Ki-67 values, no statistical difference in DFS was observed (data not shown). No correlation was found between the Ki-67 PI and mean GPS expression of tumours (Spearman *R*=0.06; *P*=0.63).

## Discussion

Cancer is regarded as a proliferative disorder, where a selective growth advantage is believed to crucially contribute to its development and progression. Supporting this concept are many studies that have shown an association between poor clinical outcome and high expression of proliferation-associated genes (Krasnoselsky *et al*, 2004; Dai *et al*, 2005; Kirschner-Schwabe *et al*, 2006).

Our study is the first to report an inverse relation between cancer malignancy and the expression of a multi-gene proliferation signature. In two independant cohorts of colon cancer patients, we observed an association between an increased recurrence risk and the reduced expression of a GPS. This finding challenges the long-held belief that rapidly dividing cancer cells are a harbinger of poor prognosis, and suggests that low proliferative activity is a biological feature of the majority of aggressive colon cancers.

The GPS we designed is highly likely to directly reflect the proliferative state of a tumour, as it was derived from both human colon crypts and an *in vitro* CRC model. All genes included in the GPS were overexpressed in actively proliferating cells of the both systems. With respect to the *in vitro* system, the comparison of exponentially growing cancer cells with contact-inhibited cancer cells has limitations; however, well established evidence indicates that many tumour cell lines maintain a variable degree of density-dependant growth suppression that is characteristic of the stationary phase (Couldwell *et al*, 1992; Steinman *et al*, 2003; Kuhn *et al*, 2004; Motti *et al*, 2005). Ten CRC cell lines were included to ensure an overall growth suppression, and only genes were considered that were altered in at least 80% of the cell lines. By the inclusion of only DE genes that overlapped with the human colon proliferation signature, the final GPS consisted of genes that strongly correlate with both colon cancer cell growth and physiological colon proliferation. Consistent with this, ontology analysis indicated that proliferation-associated genes represent the main biological theme in both the *in vitro* and the *in vivo* system.

Further evidence supporting the association of the GPS with cell proliferation stems from a considerable overlap in genes or gene families identified between our GPS and other proliferation signatures defined for tumours of the breast (Perou *et al*, 1999, 2000), ovary (Welsh *et al*, 2001), liver (Chen *et al*, 2002), acute lymphoblastic leukaemia (Kirschner-Schwabe *et al*, 2006), neuroblastoma (Krasnoselsky *et al*, 2004), lung squamous cell carcinoma (Inamura *et al*, 2005), head and neck (Chung *et al*, 2004), prostate (LaTulippe *et al*, 2002) and stomach (Hippo *et al*, 2002). Comparing these published data, Whitfield *et al* (2006) identified a core set of genes common to various proliferation signatures. As expected, these genes (MYBL2, PLK1, CDC2 and MCM genes) are also contained within our GPS (see Supplementary Table 2), reflecting the universal mechanisms that govern human cell division. Indeed, by reanalysis of public breast cancer data, our GPS was shown to perform properly in other cancer types as well. Therefore, our GPS appears to be a reliable tool for the assessment of proliferation in colonic tumours.

Application of our GPS to colon cancer patient data revealed a robust association between low proliferative activity and increased likelihood of recurrence. Firstly, the low GPS group had reduced DFS in two independant cohorts derived from different populations. Secondly, expression data from the two cohorts were obtained using two different array platforms, indicating that the observed association was not subject to methodological bias. Thirdly, reduced GPS expression in cohort A also correlated with clinico-pathological variables related to poor outcome (stage, lymphatic invasion). A possible confounding factor in our study was the chemotherapy treatment as given in 28% of cohort A patients. Exclusion of these patients from analysis had no effect on the association strength, suggesting that proliferation affects patient outcome independant of adjuvant chemotherapy.

Notably, the observed association was not independant of tumour stage. In other words, higher disease stages were enriched for slowly proliferating tumours, but tumours with high GPS expression were also present. It remains possible, however, that these fast proliferating tumours had progressed slowly before they were diagnosed at an advanced stage. It is likely that tumour stage is a better prognostic factor because the presence of lymph node and distant organ involvement is a direct manifestation of metastasis. It is noteworthy that the goal of this study was not to develop a prognostic tool based on the GPS, but to determine the nature of the relationship between proliferation and the degree of malignancy in colon cancer.

Together, the analyses we performed are all consistent with a marked effect of colon cancer proliferation on the rate of its recurrence. This is an important finding because studies using conventional proliferation markers have not been able to establish a clear-cut association between colon cancer proliferation and outcome. On account of these reported inconsistencies, we also performed DFS analysis on the basis of proliferation estimated by conventional Ki-67 labelling. Unlike the GPS, Ki-67 PI failed to separate a set of 73 CRCs into groups associated with distinct survival (Supplementary Figure 2). Although the advantage of assessing multiple genes as opposed to one proliferation marker appears obvious, additional parameters are likely to contribute to the inferior performance of the Ki-67 PI. Ki-67 positivity reflects the number of cells in cycle, rather than their cycling speed (Endl and Gerdes, 2000). Furthermore, the visual scoring of Ki-67 positive nuclei is inherently subjective, confounding correlations particularly in the case of small sample sizes (Barzanti *et al*, 2000). It is of interest, however, that a low Ki-67 PI has been associated with poor outcome in the three studies with the highest statistical power (i.e., largest sample sizes) (Allegra *et al*, 2003; Garrity *et al*, 2004; Hilska *et al*, 2005).

It is difficult to assess from our data whether slow proliferation directly enhances the ability of a colon cancer to metastasise or fast proliferation may be disadvantageous for colon cancers to acquire metastatic capacity. Although it appears intuitively logical that accelerated cell division allows for more genetic events required for progression to accumulate, fast proliferation might also have a negative impact on a tumour ability to survive and spread. For example, rapidly growing tumours may elicit a stronger and more effective immune response. Further, highly proliferative tumours may contain a lower proportion of cancer stem cells thought to undergo relatively slow divisions. Alternatively, a high level of genetic instability may increase the invasive potential of cancer cells or their resistance towards apoptosis, but may interfere with smooth replication. Also, hypoxic conditions may slow down the growth rate of tumours but could promote the onset of epithelial–mesenchymal transitions leading to invasion. Possible underlying mechanisms are currently under investigation in our laboratory.

The intriguing finding of an inverse relationship between tumour proliferative activity and disease aggressiveness suggests that fundamental biological differences exist for the mechanisms that drive disease progression in colon cancer compared with other epithelial malignancies. The delineation of the underlying processes will be important not only for the understanding of colon cancer biology, but also for the design of new therapeutic strategies. In this context, the development of alternative therapies might be a pertinent issue, given that current chemotherapeutic agents are usually designed to target colon cancer progression by killing rapidly proliferating cells.

## Figures and Tables

**Table 1:** Clinico-pathologic characteristics of two cohorts of colon cancer patients and associations with the GPS

	**Numbers**		**Cohort A**	**Cohort B**
**Parameters**	**Cohort A**	**Cohort B**	**(*P*-value)^a^**	**(*P*-value)^a^**
*Age* b				
<Mean	49	20	0.33	0.79
>Mean	59	17		
				
*Sex*				
Male	62	21	0.56	0.74
Female	46	16		
				
*Differentiation*				
Well/moderate	83	22	0.17	0.20
Poor	25	15		
				
*Disease stage*				
I	12	0	**0.015**	NA
II	64	37		
III	29	0		
IV	3	0		
				
*Vascular invasion*				
Yes	9	1	0.065	NA
No	99	36		
				
*Lymphatic invasion*				
Yes	23	3	**0.018**	1
No	85	34		
				
*Lymphocyte infiltration*				
Nil/mild	53	9	0.67	0.48
Moderate	42	17		
Prominent	13	11		
				
*Margin*				
Infiltrative	56	NA	0.84	NA
Expansive	52			
				
*Chemotherapy*				
Yes	30	0	0.08	NA
No	78	37		
				
*Recurrence at 5 years*				
Yes	24	16	**0.021**	**0.005**
No	84	21		
Total	108	37		

Abbreviations: GPS=gene proliferation signature; NA=not applicable.

a A Fisher's Exact test or Kruskal–Wallis test were used for testing association between clinico-pathologic parameters and the dichotomous GPS variable.

b Average age 68 and 63 years for cohort A and B patients, respectively. Bold numbers represent significant *P*-values.

**Table 2:** Cox regression analysis of determinants of DFS in cohort A cancer patients

	**Univariate analysis**	**Multivariate analysis^a^**	
**Parameters**	***P*-value^b^**	**HR (CI)^c^**	***P*-value**
Disease stage (I+II vs III+IV)	<0.001	4.5 (1.8–10.8)	0.001
Lymphatic invasion (− vs +)	<0.001	—	—
Vascular invasion (− vs +)	0.012	—	—
Margin (expansive vs infiltrative)	0.015	—	—
GPS expression (high vs low)	0.022	—	—

Abbreviation: DFS=disease-free survival

a Final results of Cox regression analysis using a forward stepwise method (enter limit=0.05, remove limit=0.10).

b Log-rank test *P*-value.

c Hazard ratio (HR) determined by Cox regression model; confidence interval (CI)=95%.
