# Supplementary figures and images for: Reduced expression of a gene proliferation signature is associated with enhanced malignancy in colon cancer
Source: Br J Cancer. 2008 Aug 26;99(6):966–73. doi: 10.1038/sj.bjc.6604560 (PMC2538751; doi:10.1038/sj.bjc.6604560)

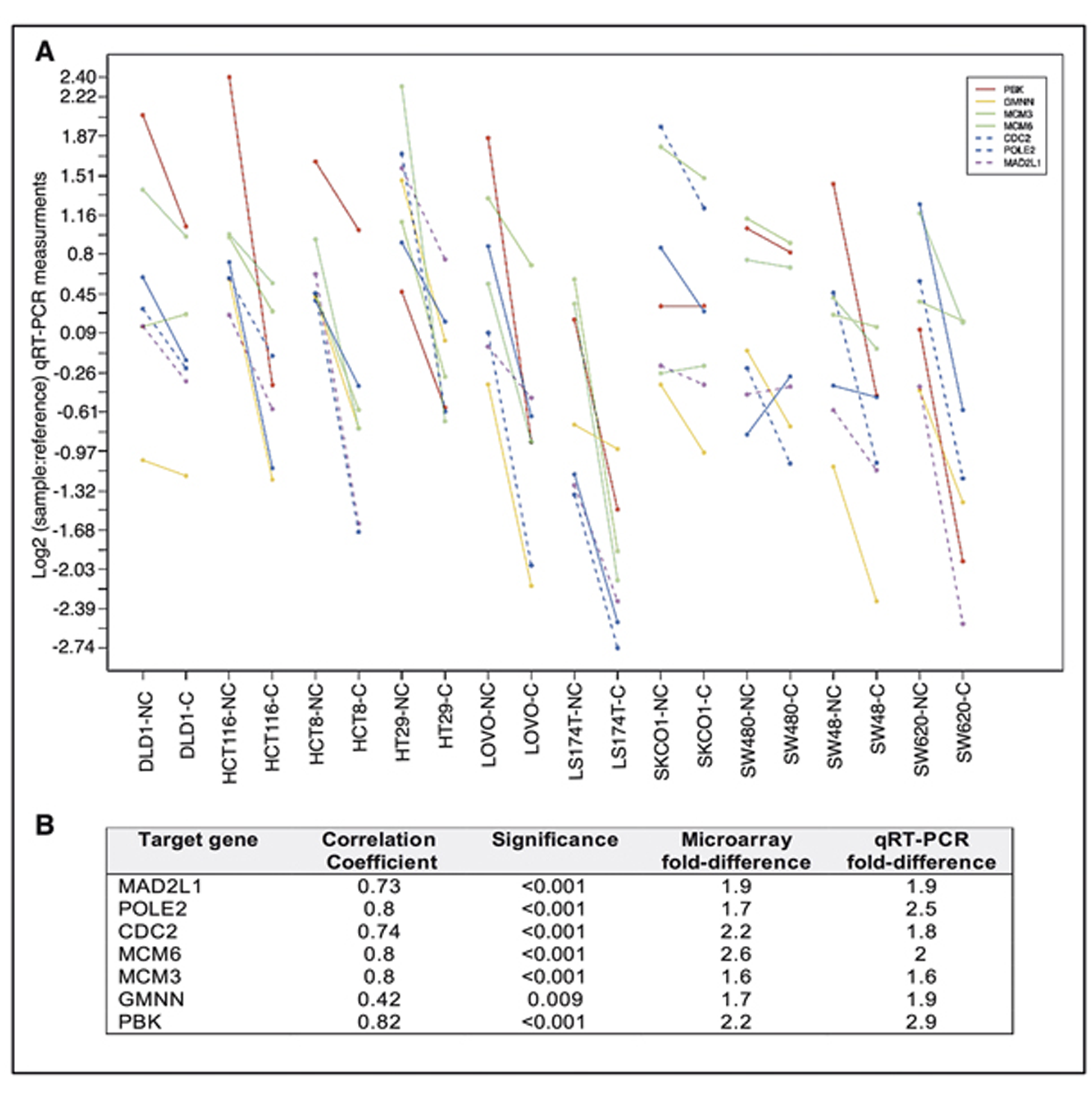

Supplement: Supplementary Figure 1 [file 6604560x1.tif]

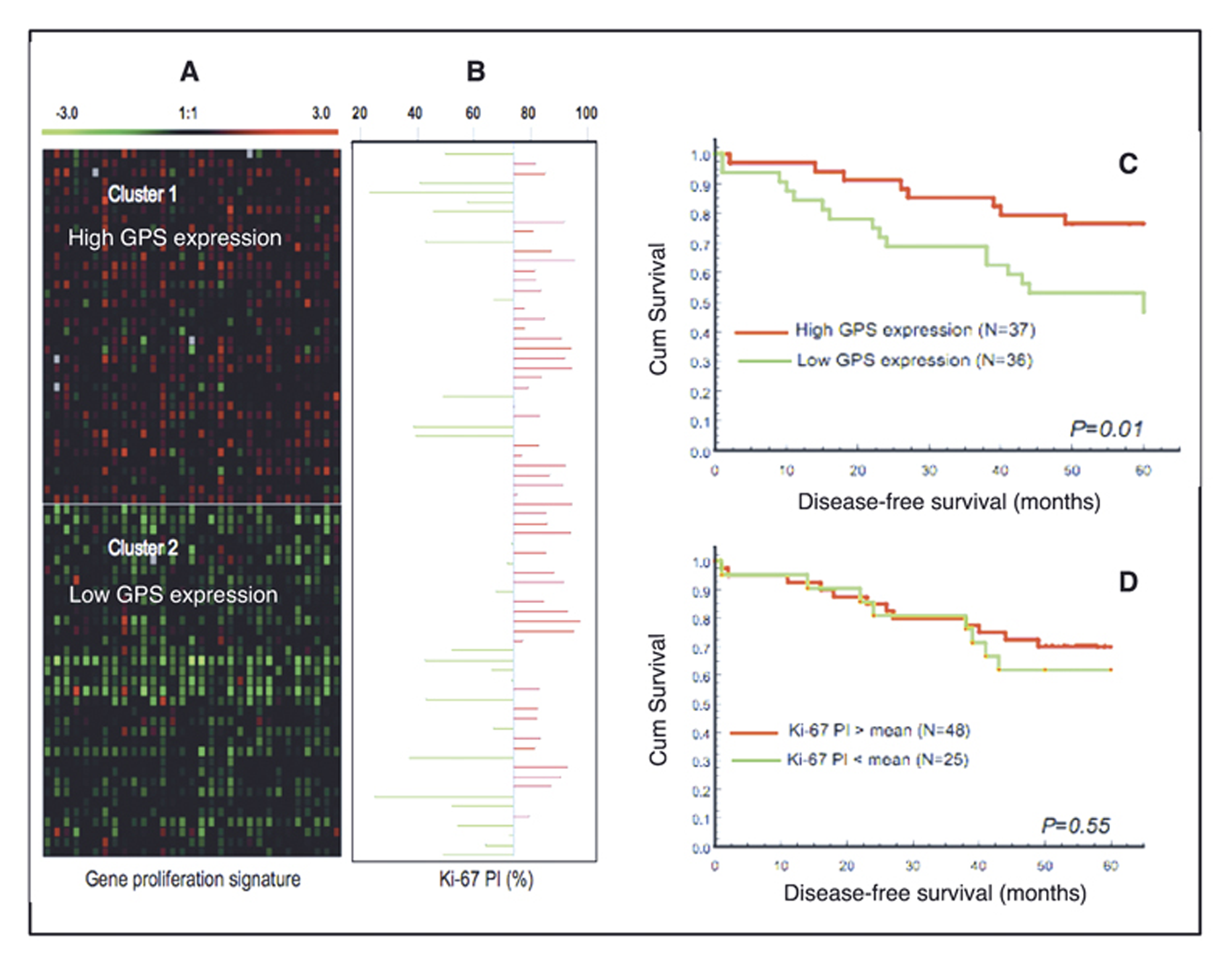

Supplement: Supplementary Figure 2 [file 6604560x2.tif]
