# Supplementary material for: Reduced expression of a gene proliferation signature is associated with enhanced malignancy in colon cancer
Source: Br J Cancer. 2008 Aug 26;99(6):966–73. doi: 10.1038/sj.bjc.6604560 (PMC2538751; doi:10.1038/sj.bjc.6604560)
Supplement: Supplementary Table 1 [file 6604560x3.doc]

Supplementary Table 1. Summary of studies on the association of proliferation indices with the colorectal cancer survival

| **Study** | **Number of patients** | **Dukes stage** | **Marker** | **Association with survival** |
| --- | --- | --- | --- | --- |
| Evans et al.^1^ | 40 | A-C | Ki-67 |  |
| Rosati et al.^2^ | 103 | B-C | Ki-67 |  |
| Ishida et al.^3^ | 51 | C | Ki-67 |  |
| Buglioni et al.^4^ | 171 | A-D | Ki-67 |  |
| Guerra et al.^5^ | 108 | A-C | PCNA | **No** association between |
| Kyzer and Gordon^6^ | 30 | B-D | Ki-67 | proliferation index and survival |
| Jansson and Sun^7^ | 255 | A-D | Ki-67 |  |
| Baretton et al.^8^ | 95 | A-B | Ki-67 |  |
| Sun et al.^9^ | 293 | A-C | PCNA |  |
| Kubota et al.^10^ | 100 | A-D | Ki-67 |  |
| Valera et al.^11^ | 106 | A-D | Ki-67 |  |
| Dziegiel et al.^12^ | 81 | NI | Ki-67 |  |
| Scopa et al.^13^ | 117 | A-D | Ki-67 | **High** proliferation index associated |
| Bhatavdekar et al.^14^ | 98 | B-C | Ki-67 | with **shorter** survival |
| Chen et al.^15^ | 70 | B-C | Ki-67 |  |
| Choi et al.^16^ | 86 | B-D | PCNA |  |
| Hilska et al.^17^ | 363 | A-D | Ki-67 |  |
| Salminen et al.^18^ | 146 | A-D | Ki-67 |  |
| Garrity et al.^19^ | 366 | B-C | Ki-67 | **Low** proliferation index associated |
| Allegra et al.^20^ | 706 | B-C | Ki-67 | with **shorter** survival |
| Palmqvist et al.^21^ | 56 | B | Ki-67 |  |
| Paradiso et al.^22^ | 71 | NI | PCNA |  |
| Neoptolemos et al.^23^ | 79 | A-C | PCNA |  |

NI, no information available

References:

1. Evans C, Morrison I, heriot A, et al. The correlation between colorectal cancer rates of proliferation and apoptosis and systemic cytokine levels; plus their influence upon survival. British Journal of Cancer 2006;94:1412-9.

2. Rosati G, Chiacchio R, Reggiardo G, et al. Thymidylate Synthase Expression, p53, bcl-2, Ki-67 and p27 in Colorectal Cancer: Relationships with Tumor Recurrence and Survival. Tumor Biology 2004;25:258-63.

3. Ishida H, Miwa H, Tatsuta M, et al. Ki-67 and CEA expression as prognostic markers in Dukes' C colorectal cancer. Cancer Letters 2004;207:109-115.

4. Buglioni S, D'Agnano I, Cosimelli M, et al. Evaluation of multiple bio-pathological factors in colorectal adenocarcinomas: Independent prognostic role of p53 and bcl-2. International Journal of Cancer 1999;84:545-552.

5. Guerra A, Borda F, Javier Jimenez F, et al. Multivariate analysis of prognostic factors in resected colorectal cancer: a new prognostic index. European Journal of Gastroenterology & Hepatology 1998;10:51-8.

6. Kyzer S, Gordon P. Determination of proliferative activity in colorectal carcinoma using monoclonal antibody Ki67. Diseases of the colon and rectum 1997;40:322-5.

7. Jansson A, Sun X. Ki-67 expression in relation to clinicopathological variables and prognosis in colorectal adenocarcinomas. APMIS 1997;105:730-4.

8. Baretton G, Diebold J, Christoforis G, et al. Apoptosis and immunohistochemical bcl-2 expression in colorectal adenomas and carcinomas. Aspects of carcinogenesis and prognostic significance. Cancer 1996;77:255-64.

9. Sun X, Carstensen J, Stal O, et al. Proliferating cell nuclear antigen (PCNA) in relation to ras, c-erbB-2,p53, clinico-pathological variables and prognosis in colorectal adenocarcinoma. International Journal of Cancer 1996;69:5-8.

10. Kubota Y, Petras R, Easley K, et al. Ki-67-determined growth fraction versus standard staging and grading parameters in colorectal carcinoma. A multivariate analysis. Cancer 1992;70:2602-9.

11. Valera V, Yokoyama N, Walter B, et al. Clinical significance of Ki-67 proliferation index in disease progression and prognosis of patients with resected colorectal carcinoma. British Journal of Surgery 2005;92:1002-1007.

12. Dziegiel P, Forgacz J, Suder E, et al. Prognostic significance of metallothionein expression in correlation with Ki-67 expression in adenocarcinomas of large intestine. Histol Histopathol 2003;18:401-7.

13. Chrisoula DS, Athanassios CT, Vicky Z, et al. Potential Role of bcl-2 and Ki-67 Expression and Apoptosis in Colorectal Carcinoma: A Clinicopathologic Study. Digestive Diseases and Sciences 2003;V48:1990-1997.

14. Bhatavdekar JM, Patel DD, Chikhlikar PR, et al. Molecular markers are predictors of recurrence and survival in patients with Dukes B and Dukes C colorectal adenocarcinoma. Diseases of the Colon & Rectum 2001;44:523-33.

15. Chen YT, Henk MJ, Carney KJ, et al. Prognostic significance of tumor markers in colorectal cancer patients: DNA index, S-phase fraction, p53 expression, and Ki-67 index. Journal of Gastrointestinal Surgery 1997;1:266-273.

16. Choi HJ, Jung IK, Kim SS, et al. Proliferating cell nuclear antigen expression and its relationship to malignancy potential in invasive colorectal carcinomas. Diseases of the Colon & Rectum 1997;40:51-9.

17. Hilska M, Collan YU, O'Laine VJ, et al. The Significance of Tumor Markers for Proliferation and Apoptosis in Predicting Survival in Colorectal Cancer. Diseases of the Colon & Rectum 2005;V48:2197-2208.

18. Salminen E, Palmu S, Vahlberg T, et al. Increased proliferation activity measured by immunoreactive Ki67 is associated with survival improvement in rectal/recto sigmoid cancer. World J Gastroenterol. 2005;11:3245-9.

19. Garrity MM, Burgart LJ, Mahoney MR, et al. Prognostic value of proliferation, apoptosis, defective DNA mismatch repair, and p53 overexpression in patients with resected Dukes' B2 or C colon cancer: a North Central Cancer Treatment Group Study. J Clin Oncol. 2004;22:1572-82.

20. Allegra CJ, Paik S, Colangelo LH, et al. Prognostic Value of Thymidylate Synthase, Ki-67, and p53 in Patients With Dukes' B and C Colon Cancer: A National Cancer Institute-National Surgical Adjuvant Breast and Bowel Project Collaborative Study. Journal of Clinical Oncology 2003;21:241-250.

21. Palmqvist R, Sellberg P, Oberg A, et al. Low tumour cell proliferation at the invasive margin is associated with a poor prognosis in Dukes' stage B colorectal cancers. British Journal of Cancer 1999;79:577-81.

22. Paradiso A, Rabinovich M, Vallejo C, et al. p53 and PCNA expression in advanced colorectal cancer: response to chemotherapy and long-term prognosis. International Journal of Cancer 1996;69:437-41.

23. Neoptolemos JP, Oates GD, Newbold KM, et al. Cyclin/proliferation cell nuclear antigen immunohistochemistry does not improve the prognostic power of Dukes' or Jass' classifications for colorectal cancer. British Journal of Surgery 1995;82:184-7.
