# Supplementary material for: Reduced expression of a gene proliferation signature is associated with enhanced malignancy in colon cancer
Source: Br J Cancer. 2008 Aug 26;99(6):966–73. doi: 10.1038/sj.bjc.6604560 (PMC2538751; doi:10.1038/sj.bjc.6604560)
Supplement: Supplementary Table 3 [file 6604560x5.doc]

**Supplementary Table 3.** Differential expression of the GPS between

pre-defined classes using the gene set comparison analysis

| **Parameters** | **Cohort A**  KS *p*-value^§^ | **Cohort B**  KS *p*-value^§^ |
| --- | --- | --- |
| **Age**  (≥ mean *vs.*< mean) | NS | NS |
| **Sex**  (male *vs.* female) | NS | NS |
| **Differentiation**  (well+moderate *vs.* poor) | NS | NS |
| **Disease stage ^‡^**  (I+II *vs.* III+IV) | ***< 1e-05*** | NS |
| **Vascular invasion**  (positive *vs.* negative) | NS | NA |
| **Lymphatic invasion**  (positive *vs.* negative) | ***0.001*** | NS |
| **Lymphocyte infiltration**  (mild +moderate *vs.* prominent) | NS | NS |
| **Margin**  (infiltrative *vs.* expansive) | NS | NA |
| **chemotherapy**  (positive *vs.* negative) | NS | NA |
| **Recurrence at 5 years**  (positive *vs.* negative) | ***< 1e-07*** | ***< 1e-07*** |

^§^Kolmogorov-Smirnov *p*-value (specified threshold of significance=0.005)

^‡^For cohort B, the GPS was compared between stage I and stage II disease.

NS; not significant

NA; not applicable
